# Knowledge, Attitudes, and Practices of Indian Dermatologists Regarding Sensitive Skin Conditions and Their Management With Colloidal Oats Among Indian Adults: A Cross-Sectional Survey

**DOI:** 10.7759/cureus.102885

**Published:** 2026-02-03

**Authors:** Abir Saraswat, Abhishek De, Narmada Matang, Priyanka Reddy, Priti Thakor, Ruchi Shah, Someshwar Rayasam

**Affiliations:** 1 Dermatology, Midland Hospital and Research Centre, Lucknow, IND; 2 Dermatology, Wizderm Skin and Hair Clinic, Kolkata, IND; 3 Dermatology, Neova Skin Clinic, Mumbai, IND; 4 Dermatology, DNA Skin Clinic, Bengaluru, IND; 5 Dermatology, JNTL Consumer Health (India) Private Limited, Mumbai, IND; 6 Medical Affairs, Kenvue, Mumbai, IND

**Keywords:** colloidal oatmeal, dermatologists, moisturizers, prescribers, sensitive skin, skin barrier

## Abstract

Objectives: This survey aimed to evaluate the knowledge, attitudes, and practices of Indian dermatologists in managing sensitive skin (SS) conditions in adult patients in India.

Methods and materials: A cross-sectional survey was conducted among 150 dermatologists, including 75 prescribers of colloidal oatmeal-based products and 75 nonprescribers, using a validated questionnaire.

Results: Most dermatologists practiced in private settings (142 (94.7%)), and 82 (54.7%) reported SS as most prevalent in individuals aged 15-29 years. Regarding triggers for SS, endogenous factors such as hormonal changes and disrupted skin barrier were identified by 130 (87.6%) and 128 (85.3%) dermatologists, respectively. Commonly reported exogenous factors included cosmetic ingredients by 122 (81.3%), environmental factors by 119 (79.3%), and lifestyle choices by 116 (77.3%). The dermatologists emphasized the importance of moisturizer use, with 147 (98%) acknowledging early moisturization as essential for SS management. Colloidal oatmeal, identified by 134 (89.3%), and ceramides, identified by 129 (86%), were the top-rated ingredients for strengthening and repairing the skin barrier.

Conclusion: The survey highlights a strong understanding among dermatologists of the importance of early moisturization and skin barrier reinforcement in managing SS. Although prescribers recognized the benefits of colloidal oatmeal-based products, awareness among nonprescribers was limited. Addressing cost barriers and the need for scientific evidence can enhance the use of oatmeal-based products in SS management.

## Introduction

Sensitive skin (SS) is a self-reported condition characterized by unpleasant sensations such as dryness, itching, or burning triggered by stimuli that typically do not elicit these reactions [1-3]. It primarily affects the face but can also involve the hands, scalp, and other areas [4]. The underlying mechanisms include sensory hyperreactivity, impaired skin barrier, and a predisposition to atopic conditions [5]. Globally, 71% of adults report some degree of skin sensitivity [6]. Research suggests that younger individuals are more prone to SS [7]. In India, 36.7% of women and 27.9% of men report having SS [8].

Environmental exposures, lifestyle factors, and endogenous factors, such as age, gender, and skin conditions, are linked to SS [3]. SS affects the quality of life (QoL), causing psychological distress, fatigue, anxiety, and sleep disturbances [9]. It is also associated with dermatological conditions, such as atopic dermatitis (AD), psoriasis, and rosacea, and disorders, including obesity and autoimmune diseases [8,10,11].

Diagnosis of SS involves sensory reactivity tests, including the lactic acid sting test (LAST), and measurements of transepidermal water loss (TEWL) [3,12]. Management of SS includes gentle skin care, moisturizers, and lipid-replenishing agents, such as ceramides [12]. Colloidal oatmeal shows promise in improving skin barrier function and reducing irritation; however, dermatologists' awareness of this remains limited [13].

In India, where climatic and genetic diversity is considerable, a clear understanding of SS definition, diagnosis, and management is essential [8]. Given the significant impact of dermatologists’ knowledge, attitudes, and practices (KAP) on patient care, the following survey was designed to explore these factors in the context of SS conditions in the Indian population. The primary objective of this study was to assess the KAP of Indian dermatologists regarding SS conditions in adult Indian patients. The secondary objectives included evaluating diagnostic approaches and common triggers of SS and examining dermatologists’ use of moisturizers, including colloidal oatmeal moisturizers, along with related prescribing patterns. Differences in KAP between dermatologists who prescribed colloidal oatmeal and those who did not were also assessed.

## Materials and methods

Study design

A cross-sectional survey of 150 dermatologists (75 prescribers and 75 nonprescribers) was conducted to assess KAP regarding SS in Indian adults. Participants were randomly selected from a national database, ensuring balanced representation of both groups. The study protocol was approved by the ACEAS - Independent Ethics Committee, Gujarat, India, dated 12 August 2024. Electronic informed consent was obtained from all the participants.

Questionnaire development and validation

The questionnaire was developed in a structured, multistep process. A targeted literature review identified key domains of SS, including triggers, clinical features, diagnostic approaches, and management strategies, with particular focus on barrier-repair agents and oatmeal-based formulations. Questionnaire items were included if supported by published evidence or clinical relevance in SS or related dermatoses applicable to routine adult dermatology practice. Items specific to pediatric populations, lacking scientific support, investigational in nature, or redundant or ambiguous were excluded. Items were framed to cover three domains: knowledge, attitudes, and practices. The expert panel consisted of key opinion leaders from the dermatology therapy area, who provided clinical insights, ensuring real-world relevance. Content validity was confirmed by the panel, and internal consistency was assessed using Cronbach's alpha (≥0.7 considered acceptable). Four experts pilot tested the questionnaire for clarity and feasibility. This was followed by final refinements and grammar checks.

Survey administration and analysis

The finalized questionnaire was administered to the selected dermatologists, and the responses were collected securely. Data were summarized using descriptive statistics. The questionnaire, presented as Appendix 1, included sections on demographics and KAP related to SS. The knowledge section covered prevalence, triggers, clinical signs, and the role of moisturizers. The attitudes section explored views on diagnosis, early moisturizer use, emollient choice, and the use of colloidal oatmeal. The practices section addressed diagnostic methods, management strategies, treatment challenges, and the use of oatmeal-based moisturizers.

Statistical analysis

Data were analyzed using R Statistical Software (v4.1.2; R Core Team, Vienna, Austria, 2021). Categorical variables are reported as frequencies and percentages, while continuous variables are reported as means and standard deviations.

## Results

Demographic information

A total of 150 dermatologists (75 prescribers and 75 nonprescribers) responded to the survey questionnaire. The overall mean age was 46.6±9.7 years, with 48.1±9.8 years among prescribers and 45.1±9.4 years among nonprescribers. The mean work experience across all participants was 19 years. The majority practiced in private hospitals or clinics (142 (94.7%)), followed by those who practiced in both private and government hospitals (6 (4%)), and those who practiced in government hospitals (2 (1.3%)).

Knowledge of dermatologists regarding SS

The most common age group experiencing SS was identified as 15-29 years, followed by 30-44 years.

Ingredients for Skin Barrier Repair

Colloidal oatmeal was identified by 134 (89.3%) dermatologists as the top ingredient, followed by ceramides, selected by 129 (86%), for strengthening and repairing the skin barrier.

**Figure 1:**
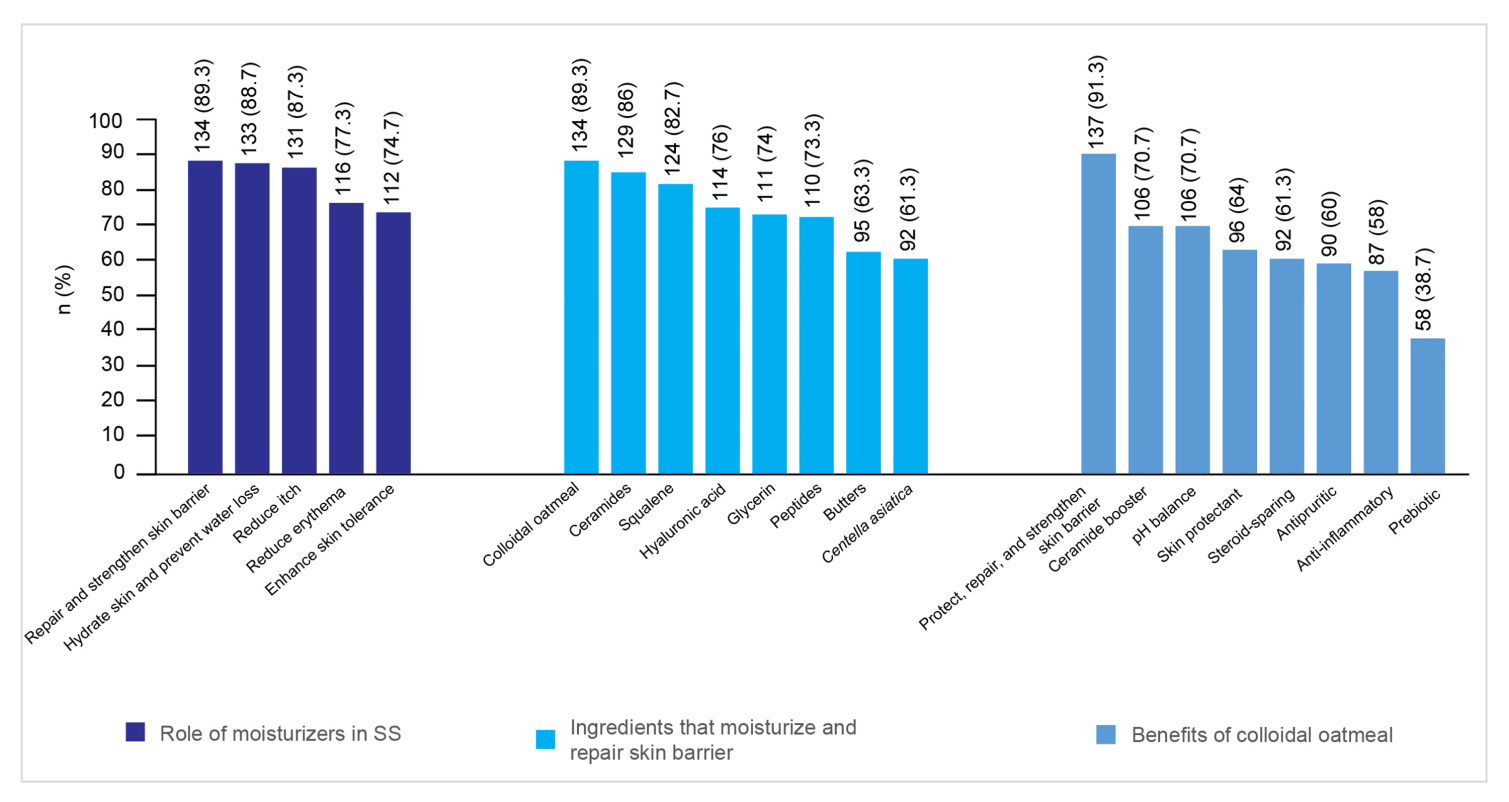
Knowledge of dermatologists regarding the role of moisturizers and colloidal oatmeal and ingredients that moisturize and repair the skin barrier SS: sensitive skin

Figure 1 depicts the knowledge of dermatologists regarding the role of moisturizers and colloidal oatmeal and ingredients that moisturize and repair the skin barrier.

Triggers of SS

**Figure 2:**
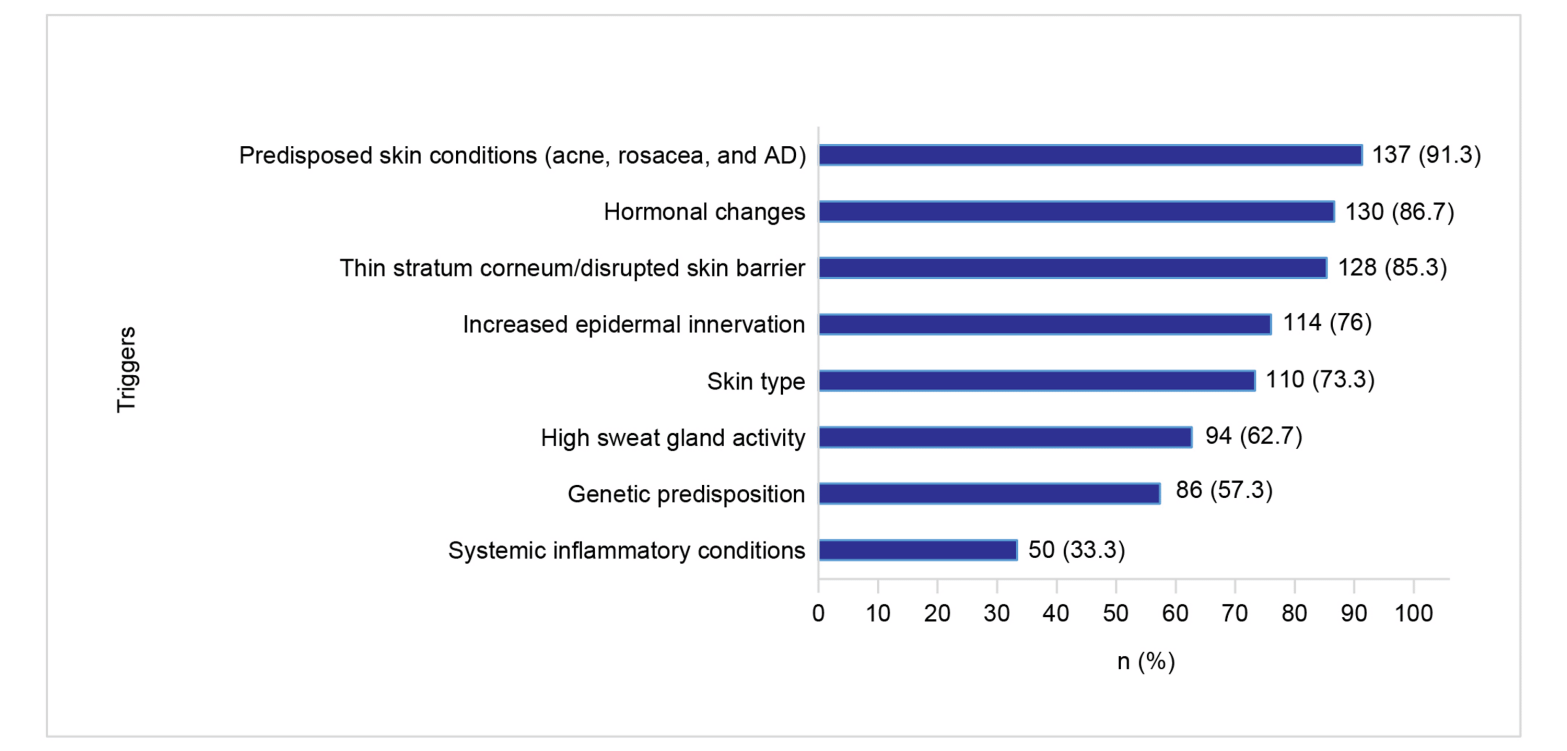
Endogenous triggers for SS AD: atopic dermatitis; SS: sensitive skin

**Figure 3:**
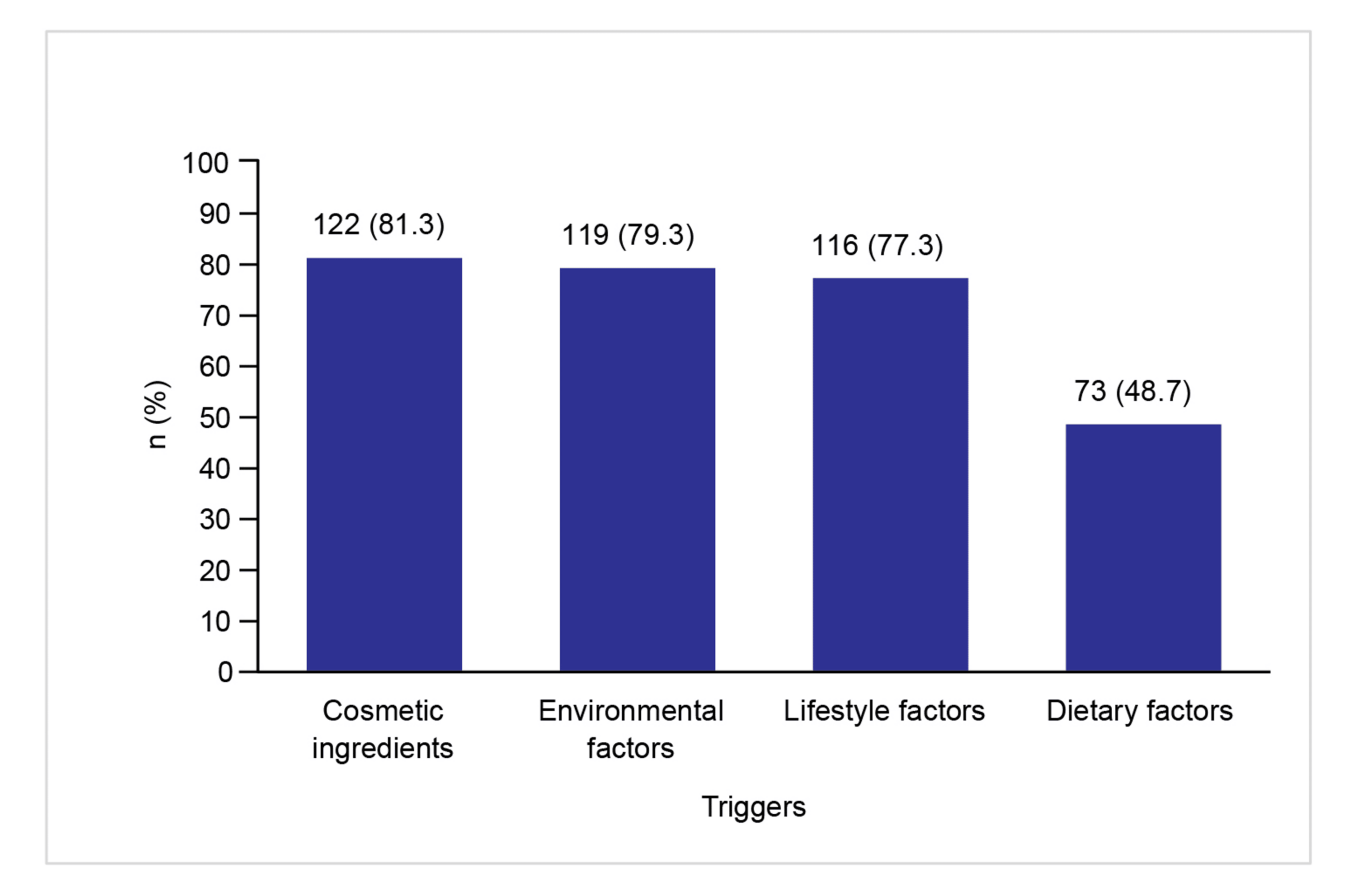
Exogenous triggers for SS SS: sensitive skin

Figures 2-3 depict the responses of dermatologists on the various endogenous and exogenous triggers for SS.

Clinical Signs

Overall, 143 (95.3%) dermatologists reported dryness, 138 (92%) itching, and 127 (84.7%) erythema as common clinical signs of SS. Scaling was reported by 121 (80.7%) dermatologists, and stinging or burning sensations by 113 (75.3%). Most recognized SS as a precursor to dermatological conditions such as AD.

Attitudes of dermatologists regarding SS

Overall, 101 (67.3%) dermatologists agreed that both clinical evaluation and diagnostic tests were more effective in diagnosing SS than either method individually. Most recognized the important role of early moisturization in preventing SS.

Parameters for Choosing Emollients

Suitability for SS was the top priority for most dermatologists (147 (98%)). Colloidal oatmeal was the most favored ingredient among prescribers (70 (93.3%)). Sebum regulation/noncomedogenic properties were considered important by 48 (64%) dermatologists. Homemade preparations were ranked the lowest by 15 (20%) dermatologists.

Among nonprescribers, 74 (98.7%) preferred dermatological testing, while 73 (97.3%) considered clinical evidence in Indian skin as the most important parameters when selecting emollients for SS.

Clinical Benefits of Moisturizers

Seventy-three (97.3%) prescribers and 75 (100%) nonprescribers valued improvement in dryness, itch, redness, and scaling with emollients the most.

Importance of Colloidal Oatmeal Moisturizers in Various Skin Conditions

In the context of managing SS conditions such as AD, 96 (64%) dermatologists rated the use of colloidal oatmeal-based moisturizers as highly important.

Around 52 (69.3%) prescribers acknowledged the role of colloidal oatmeal-based moisturizers in long-term maintenance and flare-up prevention. Additionally, 49 (65.3%) prescribers recognized the importance of colloidal oatmeal moisturizers in managing flare-ups alongside core therapy.

It was also rated effective for age-related pruritus by 50 (66.7%) and for acne by 49 (65.3%) prescribers. Its effectiveness was recognized for rosacea and allergic contact dermatitis by 43 (57.3%) prescribers, respectively, and for psoriasis by 40 (53.3%) prescribers. For iatrogenic skin conditions, such as steroid- or retinoid-induced damage, 56 (74.7%) nonprescribers reported good effectiveness.

**Figure 4:**
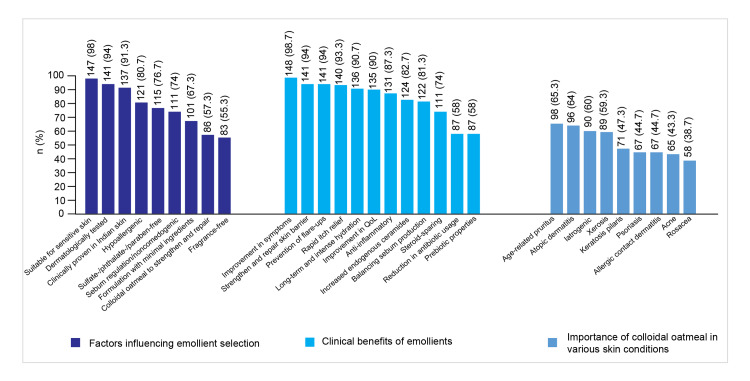
Attitudes of dermatologists regarding emollients and colloidal oatmeal QoL: quality of life

Figure 4 presents the attitudes of dermatologists toward emollients and colloidal oatmeal.

Practices of dermatologists in managing SS

Presentation of SS in Clinical Practice

In total, 77 (51.3%) dermatologists reported diagnosing SS in 30-50% of their patients, while 58 (38.7%) diagnosed it in 10-30%. Overall, 71 (47.3%) dermatologists reported that 30-50% of their adult patients present with SS symptoms during initial consultations, whereas 51 (34%) observed symptoms in 10-30% of patients. A smaller proportion (28 (18.7%)) noted prevalence between 50% and 70%, with none reporting cases exceeding 70%.

Contributing Factors and Progression in SS

Overall, 82 (54.7%) dermatologists observed that 10-30% of their adult patients with SS progressed to more severe conditions such as AD and rosacea, whereas 59 (39.3%) reported this progression in 30-50% of their patients.

Prescribers reported that the 18-29-year age group was the most affected with SS conditions. The causes were reported as acne by 58 (77.3%), hormonal imbalance by 50 (66.7%), overuse of active skin ingredients (such as glycolic acid, retinol, kojic acid, and vitamin C) by 42 (56%), and cosmetic usage by 36 (48%).

For nonprescribers, the primary reasons for SS conditions in the 18-29-year age group were acne as reported by 67 (89.3%), cosmetic usage as reported by 50 (66.7%), and rosacea as reported by 46 (61.3%).

Preferred Method to Diagnose SS

Most responses, from 132 (88%) dermatologists, indicated that the self-‍assessment questionnaire was the preferred method for diagnosing SS in clinical practice. The next preferred method, according to 64 (85.3%) nonprescribers, was measuring skin pH levels using a strip, whereas 42 (56%) prescribers favored skin barrier assessment through TEWL.

Management of SS

**Figure 5:**
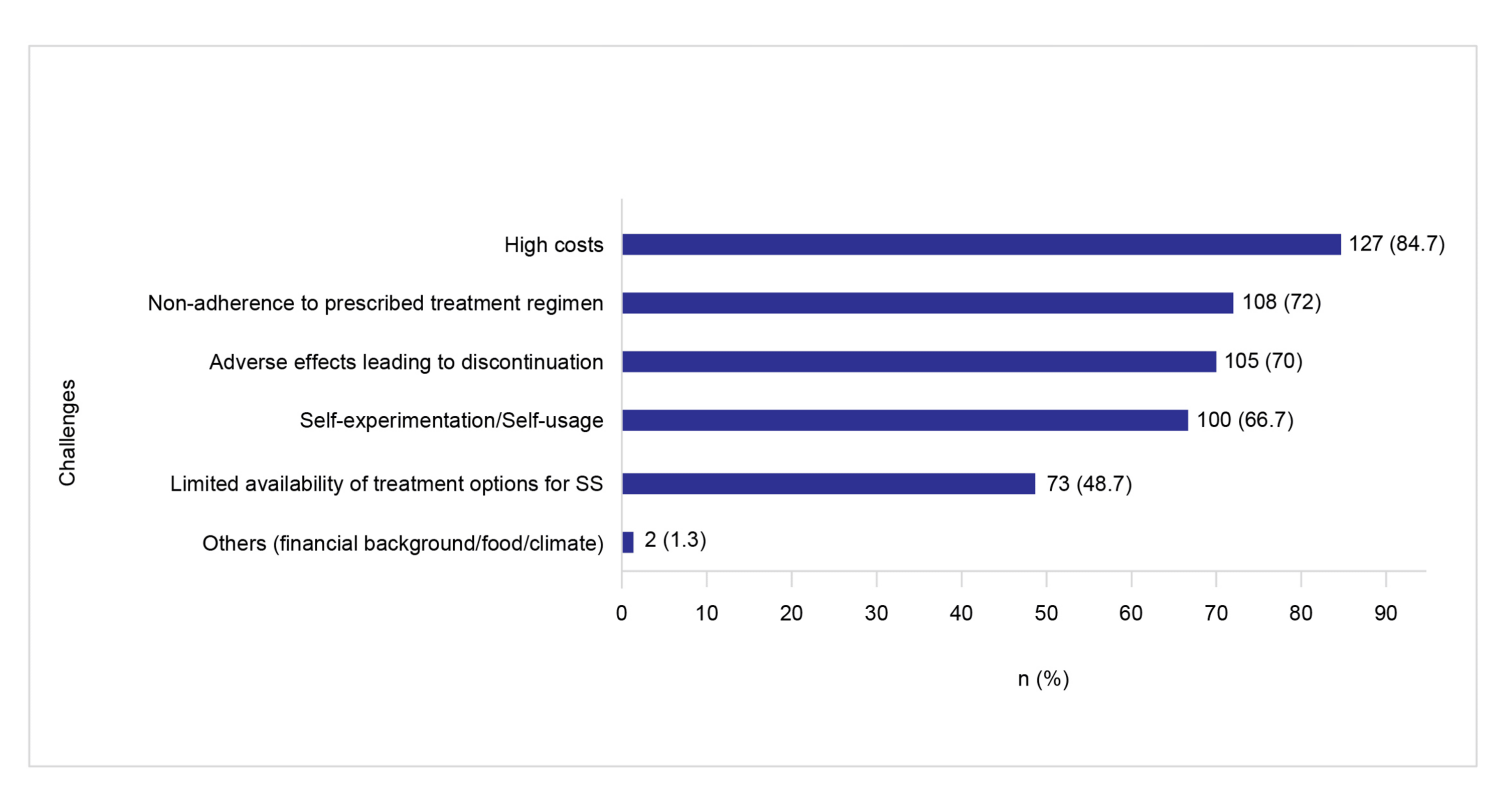
Common challenges in SS treatment SS: sensitive skin

Overall, 67 dermatologists (94.7%) recommended moisturization as the mainstay treatment for SS in 30-50% of their patients. Most dermatologists (138 (92%)) indicated that patient-reported outcomes related to symptom relief and QoL were commonly used to assess treatment effectiveness in SS conditions. The high cost of products was the most common challenge that limited treatment regimens. Patients’ nonadherence to the prescribed treatment plan and the occurrence of adverse effects due to the treatment products were other challenges, as represented in Figure 5.

Trends Among Oatmeal Prescribers

Among prescribers, 39 (52%) regularly recommended oatmeal-based moisturizers for dry skin. The use of colloidal oatmeal-based moisturizers varied, with the majority recommending it to 20-40% of patients.

About 42 (56%) prescribers observed improved QoL and reduced AD flare-ups in 30-50% of patients, and 35 (46.7%) reported decreased use of steroids, immunomodulators, and other topical agents in 30-50% of patients. Regarding clinical benefits, 25 (33.3%) prescribers reported reduced frequency and duration of active flare-ups, and an equal proportion observed improved skin barrier repair and strengthening.

**Figure 6:**
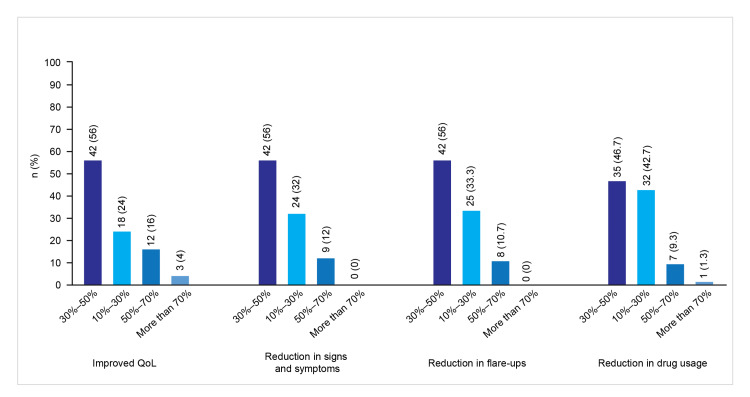
Benefits of oatmeal-based moisturizers in AD AD: atopic dermatitis; QoL: quality of life

Figure 6 presents the benefits of colloidal oatmeal in AD as reported by prescribers.

Regarding SS on the body, 46 (61.3%) prescribers recommended oatmeal-based moisturizers for >70% of their patients. In SS, on the face, 29 (38.7%) prescribed these moisturizers for more than 70% of their patients.

Around 39 (52%) prescribers recommended oatmeal-based moisturizers for dry skin; 33 (44%) for underlying conditions such as AD, psoriasis, keratosis pilaris, or dryness due to diabetes; 30 (40%) for acne or rosacea; and 28 (37.3%) for SS.

**Figure 7:**
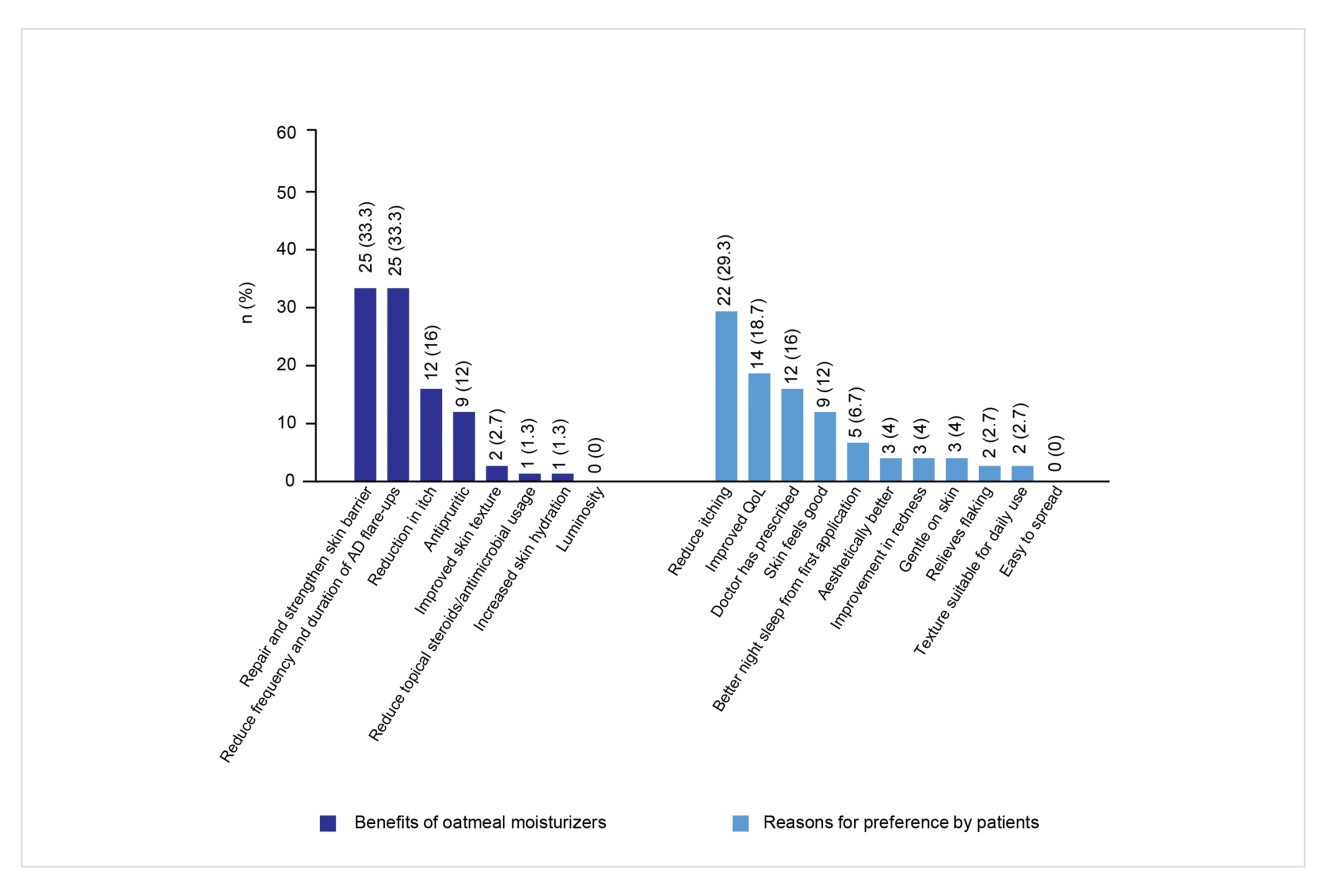
Benefits of oatmeal-based moisturizers and patient preferences AD: atopic dermatitis; QoL: quality of life

Twenty-two prescribers (29.3%) reported that patients preferred colloidal oatmeal-based moisturizers for itch relief, 14 (18.7%) noted their use for improving QoL, and 12 (16%) indicated use based on a doctor’s recommendation. Other reported reasons included a pleasant skin feel, aesthetic appeal, gentleness on the skin, and experiencing better sleep after the first application. Figure 7 presents the trends among prescribers toward colloidal oatmeal moisturizers.

Trends Among Oatmeal Nonprescribers

Overall, 40 (53.3%) nonprescribers had average knowledge of colloidal oatmeal for managing SS, followed by 27 (36%) with good knowledge, and five (6.7%) with excellent knowledge. Additionally, only 13 (17.3%) recognized their role in soothing and relieving SS symptoms.

Insufficient evidence to support the use of oatmeal-based products in patients with SS was the most common reason reported by nonprescribers (63 (84%)). This was followed by the unaffordability of the products by 17 (22.7%) and a lack of demand from patients by 13 (17.3%), while eight (10.7%) had never tried any oatmeal-based products on their patients.

**Figure 8:**
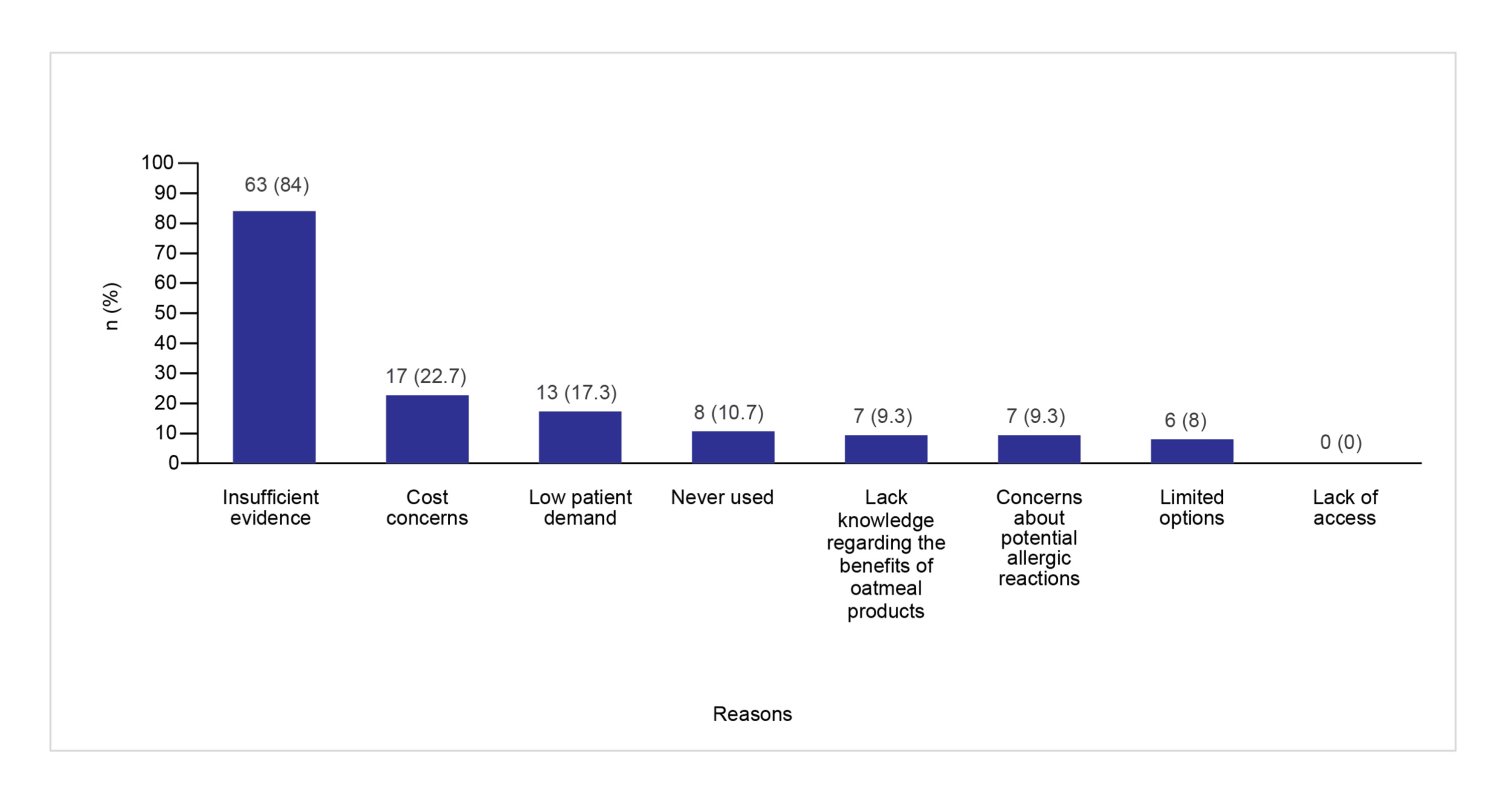
Reasons for not prescribing oatmeal-based products

About 50 (66.7%) nonprescribers expressed potential openness to prescribing or recommending oatmeal-based products for the management of SS if provided with proper scientific evidence. Figure 8 presents reasons for not prescribing oatmeal-based products among nonprescribers. 

## Discussion

SS is most prevalent among Indians aged 15-29 years, aligning with global data. The dermatologists diagnosed SS in up to 50% of patients, often during first visits. Though SS can affect any area, facial sensitivity predominates, likely due to thinner skin and frequent cosmetic exposure [7,6]. SS is influenced by emotional, dietary, and lifestyle factors. Common triggers include cosmetic ingredients, environmental exposure, and poor moisturization. SS increases the risk of dermatoses by two- to four-fold [8]. The dermatologists noted that 10-30% of SS cases progressed to AD or rosacea. SS warrants the exclusion of other dermatoses before diagnosis [14].

Assessment and diagnosis

Diagnosing SS requires both subjective and objective assessments. Objective tools include the LAST to assess stinging, skin pH, and TEWL for barrier integrity, the sodium lauryl sulfate test for sensory hyperreactivity, and patch tests for allergies. These collectively help evaluate SS and distinguish it from other dermatological conditions [5,4,15].

While LAST is widely used to identify SS, Indian dermatologists in our study favored combining clinical evaluation with diagnostic tests (101 (67.3%)). Prescribers preferred TEWL, while nonprescribers preferred skin pH. This indicates that a multifaceted approach, integrating clinical judgment and objective tools, is preferred for SS diagnosis in India [16].

Self-assessment questionnaires, ranging from simple inquiries to detailed symptom checklists, were preferred by 132 (88%) practitioners for diagnosing SS, followed by skin pH measurement by 104 (69.3%). This highlights a shift toward patient-reported outcomes, emphasizing the value of individuals' perceptions in identifying sensory and visible SS symptoms [5,10].

Management strategies in SS

Effective SS management requires a personalized approach that incorporates emollients, anti-‍inflammatory agents, photoprotection, and psychological support [15]. Moisturization is central, with 43 (57.3%) prescribers recommending it for 30-50% of their patients, and 28 (37.3%) nonprescribers recommending it for 50-70% of their patients. Prescribers reported reduced antibiotic use, better QoL, and steroid-sparing effects, while nonprescribers noted improved hydration, barrier repair, and enhanced sebum and ceramide production. Early emollient use may prevent AD or eczema [17,18]. Most participants emphasized early moisturization. Key measures to restore the barrier include avoiding aggravating factors, using preservative-free cosmetics, and adequate hydration [16]. The dermatologists prioritized suitability for SS, favoring products with minimal but validated ingredients [3].

Role of colloidal oatmeal in SS management

Several natural compounds, including hyaluronic acid, ceramides, long-chain fatty acids, and oatmeal derivatives, have been proven effective in reducing inflammation and alleviating symptoms associated with SS [16,19]. Notably, dermatologists demonstrated a stronger preference for colloidal oatmeal and ceramides, recognizing their role in barrier repair and symptom relief. Hyaluronic acid was also rated as a key ingredient in moisturizers. Of note, homemade preparations did not find favor with the dermatologists.

Colloidal oatmeal has been widely studied for its efficacy in treating dry, itchy, and irritated skin [13]. It offers multiple benefits, including inflammation reduction and skin barrier reconstruction [20], and improved skin hydration and elasticity [21]. Furthermore, its hydrating effects persist even after product discontinuation, with sustained skin hydration observed for up to two weeks in individuals with dry skin [22]. These benefits support the role of colloidal oatmeal as a valuable ingredient in SS management.

Trends among prescribers of oatmeal-based products

Oatmeal-based moisturizers were mostly recommended by prescribers for managing dry skin, followed by use for soothing symptoms of SS, including AD. They were also recommended for patients with acne or rosacea, and for addressing sensitivity triggered by active ingredient use. For SS, prescribers primarily reported reduced flare-up frequency and duration in AD, as well as improved skin barrier function, as key benefits. Some dermatologists noted antipruritic effects and decreased reliance on topical steroids and antimicrobials.

Trends among nonprescribers of oatmeal-based products

Most nonprescribers had only an average or good understanding of colloidal oatmeal in managing SS conditions. Only a small proportion recognized the role of colloidal oatmeal in soothing and relieving SS symptoms. The primary reasons for not recommending oatmeal-based products were the lack of supporting evidence, cost concerns, the lack of patient demand, and limited personal experience with these products.

Challenges in the treatment and assessment of treatment effectiveness

Patient-reported outcomes on symptom relief and QoL were the most used measures to assess SS treatment effectiveness, as reported by 75 (100%) nonprescribers and 62 (82.7%) prescribers. The key challenges included difficulty selecting suitable products, poor adherence, and high product costs. Adherence issues stemmed from the fear of side effects, unrealistic expectations, and limited patient awareness [23]. Although oatmeal-based products show promise, limited supporting evidence restricts their use [13,20-22]. Physicians expressed a willingness to recommend these products if stronger evidence emerges. Enhancing physician knowledge and dermatologist-guided regimens can improve treatment [23].

This survey demonstrates that Indian dermatologists prioritize early moisturization in sensitive skin, with 147 (98%) recognizing its importance. Colloidal oatmeal-based moisturizers were favored for their barrier-repairing and soothing properties, with prescribers reporting symptom relief and improved tolerance in up to half of patients with sensitive skin. Overall, the findings support colloidal oatmeal as a well-tolerated, barrier-focused option in management of sensitive skin, while emphasizing the need for condition-specific evidence to improve its uptake among nonprescribers.

Study limitations

Although this study offers valuable insights into the management of SS among Indian dermatologists, the survey sample size, while adequate for preliminary findings, may not fully represent the entire dermatology community. Additionally, regional biases may limit the generalizability of the results, as dermatologists in different regions may face varying environmental and patient-specific factors. Additionally, the limited use of objective diagnostic tests to confirm sensitive skin, along with the greater reliance on self-assessment questionnaires and clinical evaluation, may have influenced diagnostic consistency. Furthermore, the cost of colloidal oat-based moisturizers was identified as a barrier to routine use, potentially limiting their applicability across all patient populations. Future studies with larger, more diverse samples are needed to confirm these findings.

## Conclusions

SS is a prevalent, multifactorial condition, particularly in younger individuals. Early care and intervention, including a daily skincare routine that enhances hydration, strengthens the skin barrier, and reduces irritant susceptibility, are essential for both prevention and control. Dermatologists emphasize the importance of selecting products suited to SS, with early moisturization playing a vital role in preventing the onset and progression of SS.

Colloidal oatmeal is widely recognized for its ability to repair and protect the skin barrier, reduce dryness by locking in moisture, and soothe irritation and inflammation. Its regular use can improve overall skin quality and help manage SS, including flare-ups seen in conditions such as AD. However, the high cost of specialized oat-based moisturizers remains a significant barrier to wider adoption. Educating physicians on product characteristics and addressing factors such as cost and adherence can further improve treatment outcomes and prevention strategies.

## Appendices

Knowledge, attitude, and practices (KAP) survey questionnaire

KAP among Indian dermatologists in diagnosing and managing sensitive skin conditions in Indian adults.

A.     Demographic information

Age: Click or tap here to enter text. (in years)

Work experience: Click or tap here to enter text. (in years)

Subspecialty: Click or tap here to enter text.

Practice: ☐ Government hospital     ☐ Private hospital/clinic     ☐ Both

Practicing state: Click or tap here to enter text.

Practicing area (city): Click or tap here to enter text.

B.      Knowledge of dermatologists regarding sensitive skin

1.       Sensitive skin is frequently reported in which age group?

☐15-29 years

☐30-44 years

☐45-59 years

☐≥60 years

2.       Which of the following are the factors that trigger sensitive skin in adults? (Select all that apply.)

☐Environmental factors

☐Skin type

☐Lifestyle factors

☐Cosmetic ingredients

☐Dietary factors

☐Genetic predisposition

3.   Which of the following are the endogenous factors that trigger sensitive skin in adults? (Select all that apply.)

☐High sweat gland activity

☐Thin stratum corneum/disrupted skin barrier

☐Increased epidermal innervation

☐Hormonal changes

☐Predisposed skin conditions, such as acne, rosacea, and atopic dermatitis

☐Systemic inflammatory conditions, such as inflammatory bowel syndrome or lactose intolerance

4.   Which of the following is the most common trigger for sensitive skin reactions?

☐Harsh weather conditions

☐Overuse of active ingredients

☐Sun exposure

☐Inadequate moisturization

☐Exposure to allergens

☐Fragrances

☐Diet

5.       Clinical signs and symptoms of sensitive skin may include: [Multiple answers; please select all that apply.]

☐Itching

☐Stinging or burning of skin

☐Dryness

☐Skin pain

☐Scaling

☐Erythema

☐Stretchy or tight skin

6.       Are you aware of sensitive skin as a precursor to various dermatological conditions including atopic dermatitis based on scientific literature?

☐Yes

☐No

☐Do not know

7.       Do you know what the primary role of moisturizers is in the management of sensitive skin and associated skin diseases? (Select all that apply.)

☐To hydrate the skin and prevent excess water loss from the skin

☐To repair, restore, and strengthen the skin barrier

☐To enhance skin tolerance

☐To reduce erythema of the skin

☐To reduce itch

8.       Do you know which of the following ingredients in a moisturizer strengthen and repair the skin barrier? (Select all that apply.)

☐Hyaluronic acid

☐Colloidal oatmeal

☐Ceramides

☐Glycerin

☐Butters

☐Centella asiatica

☐Squalene

☐Peptides

9.       Which benefit of colloidal oatmeal are you aware of in sensitive skin? (Select all that apply.)

☐Protects, repairs, and strengthens skin barrier

☐Acts as a ceramide booster (helps in endogenous ceramide production by 71%)

☐Anti-inflammatory

☐pH balance

☐Prebiotic

☐Antipruritic

☐Steroid sparing

☐Approved by the USFDA as a skin protectant

C.     Attitude of dermatologists regarding sensitive skin

10.   To confirm sensitive skin diagnosis, how important do you feel diagnostic tests are as opposed to depending solely on the clinical evaluation?

**Table 1:** -

Parameters	Level of importance assigned				
	Very high	High	Medium	Low	Very low
	1	2	3	4	5
Diagnostic tests					
Clinical evaluation alone					
Both clinical evaluation and diagnostic tests					

11.   Do you feel that early intervention of moisturizers can prevent sensitive skin conditions?

☐Yes

☐No

☐Do not know

12.   While choosing an emollient to address sensitive skin conditions, what is the level of importance of the following parameters: (Single answer for each row)

**Table 2:** -

Parameters	Level of importance assigned				
	Very high	High	Medium	Low	Very low
	1	2	3	4	5
Suitable for sensitive skin					
Dermatologically tested					
Clinically proven in Indian skin					
Fragrance free					
Sulfate/phthalate/paraben free					
Hypoallergenic					
Specialized emollient such as colloidal oatmeal to strengthen and repair skin barrier					
Sebum regulation/non-comedogenic					
Formulation with minimal ingredients					

13.   What is the level of importance of the ingredients mentioned below in a moisturizer to address the pathology of atopic skin condition and help not only in moisturization but also in skin barrier repair? [Single answer for each row]

**Table 3:** -

Ingredients	Level of importance assigned				
	Very high	High	Medium	Low	Very low
	1	2	3	4	5
Hyaluronic acid					
Colloidal oatmeal					
Ceramide					
Glycerin					
Butters such as shea butter and cocoa butter					
Centella asiatica					
Peptides					
Ghee					
Aloe vera					
Homemade preparations					

14.   What is the level of importance of the clinical benefits mentioned below while choosing an emollient to address sensitive skin conditions, including eczema and atopic dermatitis? [Single answer for each row]

**Table 4:** -

Clinical superiority claim	Level of impact assigned				
	Very high	High	Medium	Low	Very low
	1	2	3	4	5
Improvement in dryness, itch, redness, and scaling					
Improvement in quality of life					
Strengthens and repairs the skin barrier					
Increased endogenous ceramide production					
Prevention of flare-ups					
Anti-inflammatory properties leading to the reduction in the itch–scratch cycle					
Steroid-sparing effect					
Reduction in antibiotic usage					
Prebiotic properties leading to improvement in microbial diversity					
Rapid itch relief					
Long-term and intense hydration					
Balancing sebum production					

15.   What is the level of importance of the usage of a colloidal oatmeal-based moisturizer alongside core therapy in active flare-ups management in atopic dermatitis? [Single answer for each row]

**Table 5:** -

		Level of importance assigned
1	High	m
2	Medium	m
3	Low	m
4	Do not know	

16.   What is the level of importance of the long-term usage of a colloidal oatmeal-based moisturizer as a single therapy in the maintenance phase for atopic dermatitis to prevent subsequent flare-ups? [Single answer for each row]

**Table 6:** -

		Level of importance assigned
1	High	
2	Medium	
3	Low	

17.   What is the level of importance of a colloidal oatmeal-based moisturizer in the management of sensitive skin conditions? [Single answer for each row]

**Table 7:** -

Sensitive skin conditions	Level of importance assigned				
	Very high	High	Medium	Low	Very low
	1	2	3	4	5
Atopic dermatitis					
Acne					
Rosacea					
Psoriasis					
Keratosis pilaris					
Iatrogenic steroids (TSDF, i.e., topical steroid-damaged face), retinoids, other drugs such as statins, oncology treatment related, etc.					
Allergic contact dermatitis					
Xerosis					
Age-related pruritus					

D.     Clinical practices of dermatologists regarding sensitive skin

18.   What percentage of your adult patients report sensitive skin symptoms during initial consultations?

☐10%-30%

☐30%-50%

☐50%-70%

☐More than 70%

19.   What percentage of your patients are diagnosed with sensitive skin?

10%-30%

30%-50%

50%-70%

More than 70%

20.   What percentage of your adult patients progress from sensitive skin to much more pronounced skin conditions, such as atopic dermatitis and rosacea?

☐10%-30%

☐30%-50%

☐50%-70%

☐More than 70%

21.   In your clinical practice, what is the most common age group for these sensitive skin conditions?

**Table 8:** -

Reason	Age group			
	18–29 years	30–44 years	45–59 years	≥60 years
	Dry skin				
Acne				
Rosacea				
Underlying diseases, such as atopic dermatitis, psoriasis, and keratosis pilaris				
Cosmetic usage				
Overuse of active skin ingredients, such as glycolic acid, retinol, kojic acid, and vitamin C				
Hormonal imbalance				
Stress and lifestyle: smoking/alcohol				
Pregnancy				

21.   In your clinical practice, what is your preferred method to diagnose sensitive skin? (Select all that apply.)

☐Lactic acid stinging test (LAST)

☐Self-assessment questionnaire

☐Measurement of skin pH levels using pH strips

☐Irritant reactivity test with sodium lauryl sulfate

☐Skin barrier assessment by TEWL measurement

☐Skin microbiome testing

☐Patch testing

☐None of the above

22.   In your clinical practice, what percentage of patients do you recommend moisturization as the mainstay of treatment in sensitive skin?

☐10%-30%

☐30%-50%

☐50%-70%

☐More than 70%

23.   How do you assess the effectiveness of the treatment provided for sensitive skin conditions in your patients? (Select all that apply.)

☐Based on patient-reported outcomes related to symptom relief and QOL

☐Based on improvement of visible signs, such as erythema and scaling

☐Based on the result of the skin tests/machine-based measurements such as TEWL

24.   What is the most common challenge you face in your clinical practice in providing optimal care for patients with sensitive skin? (Select all that apply.)

☐Non-adherence of the patients to the prescribed treatment regimen

☐Adverse effects caused by the prescribed treatment products leading to discontinuation

☐High cost of the prescribed products that limit the treatment regimen

☐Limited availability of treatment options tailored for sensitive skin

☐Self-experimentation/self-usage

☐Any other reason, please specify ----------------------------

25.   Do you prescribe/recommend oat-based moisturizers for your patients with sensitive skin?

☐Frequently

☐Less frequently

☐Occasionally

☐Never

If yes (oatmeal prescribers)

a) In your clinical practice, in what percentage of eligible patients do you recommend colloidal oat-based moisturizers?

b) In your clinical practice, which of the following benefits have been observed with the usage of a colloidal oatmeal moisturizer in patients with sensitive skin conditions?

☐Repairs and strengthens skin barrier as demonstrated by the improvement in clinical signs and symptoms

☐Reduces the frequency and duration of active flare-ups in atopic dermatitis

☐Reduces topical steroids and antimicrobial usage

☐Antipruritic and thus breaks the itch-scratch cycle

☐Reduction in itch

☐Improved texture of skin

☐Luminosity

☐Increase in skin hydration levels

c) In your clinical practice, to what extent are oat-based moisturizers effective in soothing and relieving symptoms of sensitive skin compared to non-oat-based moisturizers?

☐Highly effective

☐Moderately effective

☐Minimally effective

☐Similar, better, or worse than non-oat-based products

d)      In your clinical practice, in what percentage of atopic dermatitis patients is there improvement in QoL?

☐10%-30%

☐30%-50%

☐50%-70%

☐More than 70%

e)       In your clinical practice, improvement in clinical signs and symptoms has been observed in what percentage of atopic dermatitis patients using colloidal oatmeal moisturizer?

☐10%-30%

☐30%-50%

☐50%-70%

☐More than 70%

g)      In your clinical practice, reduction in frequency and duration of active flare-ups has been observed in what percentage of atopic dermatitis patients using colloidal oatmeal moisturizer?

☐10%-30%

☐30%-50%

☐50%-70%

☐More than 70%

h)      In your clinical practice, reduction in immunomodulators (tacrolimus), topical steroids, anti-pruritic, calming agents and antimicrobial usage has been observed in what percentage of atopic dermatitis patients using colloidal oatmeal moisturizer?

☐10%-30%

☐30%-50%

☐50%-70%

☐More than 70%

i)        Based on location, in what percentage of patients do you prescribe colloidal oat-based moisturizers? (Select one answer only for face and body.)

**Table 9:** -

Percentage			
	Face sensitivity	Body sensitivity	
10%–30%			
30%–50%			
50%–70%			
>70%			

j)        In which of the following conditions do you recommend oat-based moisturizers (Select all that apply.)

☐Acne/rosacea

☐Dry skin

☐Sensitive skin due to cosmetic usage/active skin ingredients usage

☐Hormonal imbalance

☐Underlying disease conditions such as AD, psoriasis, KP, and dryness due to diabetes

☐Post-procedural cases

☐Xerosis

k)      Patients prefer colloidal oat-based moisturizers because:

☐Reduces itching

☐Skin feels good

☐Doctor has prescribed

☐Aesthetically better accepted

☐Easy to spread

☐Improvement in redness

☐Improved quality of life

☐Relieves flaking

☐Texture suitable for daily use

☐Better night’s sleep from first application

☐Gentle on skin

If no (oatmeal non-prescribers)

a)       How would you rate your knowledge regarding scientific data on the efficacy of colloidal oatmeal in managing sensitive skin?

☐Excellent

☐Good

☐Average

☐Poor

☐Do not know

b)       Do you believe that colloidal oatmeal can aid in soothing and relieving symptoms of sensitive skin?

☐Yes

☐No

☐Not sure

c)       What are your reasons for not prescribing oatmeal-based products to your patients with sensitive skin? (Select all that apply.)

☐I lack knowledge regarding the benefits of oatmeal products.

☐I am concerned about potential allergic reactions.

☐My patients do not demand oatmeal products.

☐I do not find sufficient evidence to support its use in patients with sensitive skin.

☐I do not find oatmeal products to be affordable to prescribe to my patients.

☐I have never tried oatmeal products in my patients.

☐Limited options for oatmeal-based preparations

☐Lack of access

d)       Would you be willing to prescribe/recommend oat-based products, if you were made aware of the right scientific evidence for managing sensitive skin?

☐Yes

☐No

☐Maybe
